# Central venous catheter placement: where is the end of the line?

**DOI:** 10.1186/cc10815

**Published:** 2012-03-20

**Authors:** K Tizard, I Welters

**Affiliations:** 1 Royal Liverpool University Hospital, Liverpool, UK

## Introduction

There is still controversy regarding safe placement of central venous catheters (CVCs) as to where the tip should lie to avoid mechanical complications whilst maintaining effective use [1,2]. The carina has previously been suggested as a useful landmark to avoid intracardiac placement and its associated risks, and also that the catheter tip should lie within the superior vena cava parallel to its walls [1,2]. However, this has been disputed and there remains no consensus as to optimal tip placement. To gauge our current practice we performed a retrospective review of CVCs placed via the internal jugular or subclavian route in intensive care patients to assess where CVC tips were placed.

## Methods

We retrospectively reviewed the chest radiographs of 197 consecutive intensive care patients admitted on and before 30 June 2011. A total of 101 patients had evidence of 137 new CVCs. For each new catheter the Picture Archiving & Communication System was used to record the tip position (after any repositioning) in relation to the carina and the degree of angulation from the vertical.

## Results

Twenty-five per cent (34/137) of all catheter tips lay >10 mm below the carina, therefore potentially increasing the likelihood of intracardiac placement. This was reduced for left-sided catheters (6/37; 16%). All right-sided catheters lay at an angle <30°. However, 38% (14/37) of left-sided catheters had not crossed the midline, and 59% (22/37) lay at an angle >30° to the vertical. Only 11% (4/37) of left-sided catheters had crossed the midline and lay at an angle of <30°, and all of these lay below the level of the carina. No immediate complications of insertion were identified. See Table 1.

**Table 1:** Site of CVC insertion (***n ***= 137)

	Internal jugular	Subclavian
Right	95	5
Left	32	5

## Conclusion

There was a wide variation of catheter tip placements accepted without re-positioning. Left-sided catheter tips are more at risk of less precise (and thus potentially nonoptimal) placement. Our results indicate that a clearer placement strategy is required.
